# Severe Cytokine Release Syndrome After CAR T Cell Therapy in a Pediatric Patient With Relapsed ALL

**DOI:** 10.1155/crom/6680945

**Published:** 2026-02-13

**Authors:** Abdulrahman Alotaibi, Noura Alajmi, Mohammed Alnuhait

**Affiliations:** ^1^ Department of Pharmaceutical Care Services, King Abdulaziz Medical City, Riyadh, Saudi Arabia, ngha.med.sa; ^2^ Department of Pharmacy Practice, College of Pharmacy, King Saud bin Abdulaziz University for Health Sciences, Riyadh, Saudi Arabia, ksau-hs.edu.sa; ^3^ King Abdullah International Medical Research Center, Riyadh, Saudi Arabia, kaimrc.med.sa; ^4^ Department of Clinical Pharmacy, College of Pharmacy, Shaqra University, Al-Dawadmi, Saudi Arabia, su.edu.sa

**Keywords:** ALL, anakinra, CAR-T, cytokine release syndrome, emapalumab, intensive care, pediatrics

## Abstract

**Background:**

Cytokine release syndrome (CRS) is a potentially life‐threatening complication of chimeric antigen receptor (CAR) T cell therapy, particularly in pediatric relapsed acute lymphoblastic leukemia (ALL).

**Case Presentation:**

A 7‐year‐old boy with early bone‐marrow relapse of hypodiploid ALL received anti‐CD19 CAR T cells and developed severe CRS with persistent fever, hypotension, hypoxemia, encephalopathy, and multiorgan dysfunction requiring pediatric intensive care.

**Management:**

He received tocilizumab, high‐dose dexamethasone, continuous intravenous (IV) anakinra, and emapalumab, plus advanced supportive care (mechanical ventilation, vasopressors, and continuous renal replacement therapy). Sequential, multiagent immunomodulation was associated with transient hemodynamic stabilization.

**Conclusion:**

This case highlights practical bedside sequencing and escalation for refractory pediatric CRS and suggests a potential role for continuous IV anakinra and emapalumab when first‐line therapy is inadequate.

**Learning Points**

• Early consideration of continuous intravenous (IV) anakinra may be beneficial in selected cases of refractory CRS. Refractory CRS following pediatric CAR T cell therapy may require escalation beyond interleukin‐6 (IL‐6) blockade within a multidisciplinary intensive care setting.
• Continuous IV anakinra and interferon‐*γ* blockade represent emerging therapeutic options for selected cases of treatment‐refractory CRS, though evidence remains limited.
• Severe CRS may progress despite aggressive immunomodulatory therapy, highlighting the need for improved predictive markers and standardized escalation strategies.

## 1. Introduction

Acute lymphoblastic leukemia (ALL) is the most prevalent malignancy in children, accounting for approximately 25% of all childhood cancers. With the advent of risk‐adapted, multiagent chemotherapy regimens, the prognosis for pediatric ALL has significantly improved, with cure rates exceeding 85% in many high‐income settings [1]. However, relapse, particularly early bone‐marrow relapse, remains a formidable clinical challenge, often associated with poor outcomes and limited therapeutic options. This is especially true in cases involving unfavorable cytogenetic features such as hypodiploidy, which are linked to chemoresistance and inferior survival rates. Chimeric antigen receptor (CAR) T cell therapy has emerged as a revolutionary treatment for relapsed or refractory B cell ALL, offering a novel mechanism of action by reprogramming a patient′s own T lymphocytes to target and eliminate malignant B cells expressing CD19. Clinical trials have demonstrated impressive remission rates, leading to regulatory approvals and incorporation of this therapy into treatment algorithms for pediatric relapsed ALL [2]. However, the success of CAR T cell therapy is tempered by its potential to induce serious complications, the most significant of which is cytokine release syndrome (CRS). CRS is a systemic inflammatory response triggered by the activation and proliferation of CAR T cells and the subsequent massive release of proinflammatory cytokines such as IL‐6, interleukin‐1 (IL‐1), and interferon‐gamma (IFN‐*γ*). Clinically, CRS can range from mild flu‐like symptoms to life‐threatening conditions involving vascular leakage, hypotension, coagulopathy, organ dysfunction, and neurological complications. In pediatric patients, the severity of CRS can escalate rapidly, necessitating early recognition and prompt, coordinated multidisciplinary intervention [3]. Current management strategies for CRS involve immunomodulatory agents such as tocilizumab, a monoclonal antibody targeting the IL‐6 receptor, which has become the cornerstone of treatment. Corticosteroids are often employed for more severe or refractory cases [4]. In the present case, high‐dose frequent dexamethasone was selected instead of pulse methylprednisolone based on institutional CRS management protocols and multidisciplinary consensus, balancing inflammatory control with preservation of CAR T cell activity. In recent years, the role of additional agents such as anakinra, an IL‐1 receptor antagonist, and emapalumab, which targets IFN‐*γ*, has been explored, particularly in cases unresponsive to first‐line therapies [5]. These emerging interventions offer promising avenues for improving outcomes in patients with high‐grade CRS, though clinical experience remains limited and largely anecdotal. Current international guidelines recommend IL‐6 blockade as first‐line therapy for CRS, with corticosteroids reserved for severe or refractory cases; however, evidence guiding escalation beyond IL‐6 inhibition in pediatric patients remains limited [6]. We present the case of a 7‐year‐old boy with early bone‐marrow relapse of ALL and high‐risk cytogenetics, who developed severe CRS following CAR T cell therapy. His clinical course required an intensive, multiagent management strategy incorporating tocilizumab, anakinra, dexamethasone, and emapalumab, alongside advanced supportive measures. This report offers a detailed account of his treatment journey, highlighting therapeutic decision‐making and outcomes, and is aimed at contributing meaningful insights to the evolving literature on CRS management in pediatric patients receiving CAR T cell therapy.

## 2. Case Presentation

### 2.1. Patient Information

A 7‐year‐old boy (22.1 kg) with hypodiploid early bone‐marrow relapse ALL was diagnosed on October 16, 2022. He was initially treated under the very high risk (VHR) arm of the COG AALL1131 protocol and achieved remission by November 28, 2022. Unfortunately, an isolated bone‐marrow relapse occurred on January 22, 2024, during the maintenance phase. He was then treated with the COG AALL1331 protocol, concluding with a FLAG chemotherapy cycle initiated on March 17, 2024. Following early isolated bone‐marrow relapse during maintenance therapy, salvage treatment with the FLAG regimen was administered prior to referral for CAR T cell therapy.

### 2.2. Clinical Course and Management

**Figure 1:**
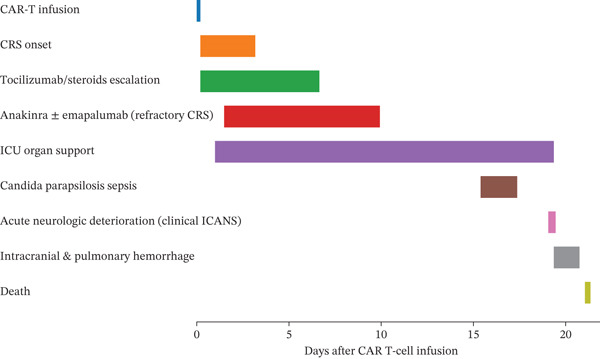
Clinical course following CAR T cell therapy (time‐scaled).

Following FLAG chemotherapy, the patient had a high disease burden, with bone marrow blasts comprising 89.2% at the time of CAR T cell infusion. No extramedullary disease was identified on clinical or radiologic assessment. The patient received anti‐CD19 CAR T cell therapy (tisagenlecleucel, tisa‐cel; Kymriah) on May 26, 2024. Shortly thereafter, he developed persistent high‐grade fever, for which empiric meropenem was started. Fever developed within 4 h following CAR T cell infusion. As desaturation ensued, he was administered tocilizumab and vancomycin. The clinical condition worsened, marked by hypotension, tachycardia, and hallucinations, leading to pediatric intensive care unit (PICU) admission. Dexamethasone and epinephrine were initiated and titrated based on blood pressure. Tocilizumab was initiated at a dose of 260 mg intravenously on Day 0 at the time of PICU admission. Due to persistent and recurrent CRS manifestations, additional doses were administered, for a total of five doses during the clinical course. Dexamethasone was started concurrently at 10 mg intravenously every 12 h and was subsequently escalated to every 8 h and then every 6 h in response to worsening hemodynamic instability and hypoxemia. Given refractory CRS with hyperinflammatory features, anakinra was introduced and escalated from intermittent dosing to continuous intravenous (IV) infusion at a total daily dose of 300 mg (13.6 mg/kg/day). Despite multiple interventions, the patient experienced recurrent desaturation (80%–85%) and tachycardia (HR up to 175 bpm). High‐flow nasal oxygen and anakinra were also initiated. Due to ongoing hemodynamic instability, dexamethasone was increased to every 6 h, and norepinephrine was added to augment vasopressor support. A fourth dose of tocilizumab and increased anakinra (2 mg/kg every 6 h) were administered. Fluid overload necessitated a continuous furosemide infusion. Subsequently, the patient developed worsening mental status, peripheral edema, and tachycardia. Milrinone was initiated, followed by elective intubation due to respiratory fatigue. Continuous renal replacement therapy (CRRT) and an anakinra infusion were started. The patient developed seizures, managed with levetiracetam. Noncontrast brain computed tomography performed 2 days after CAR T cell infusion showed no acute intracranial pathology. Cerebrospinal fluid analysis was not performed due to clinical instability. Antimicrobial therapy was escalated to meropenem and linezolid. He developed melena and mucosal bleeding, managed with cryoprecipitate and fresh frozen plasma. Coagulation management followed institutional protocols, with transfusion targets including hemoglobin > 80 g/L, platelets > 30,000/*μ*L, and fibrinogen levels maintained around 1.5 g/L using cryoprecipitate. Given refractory CRS and multiorgan dysfunction, emapalumab (1 mg/kg) was initiated. The dexamethasone frequency was reduced, and anakinra dosing was tapered over the following days. Clinical improvement followed: Meropenem was de‐escalated, feeding resumed, ventilatory support was weaned, and inotropes were discontinued. CRRT was stopped 3 days later, and the patient continued to stabilize. After initial resolution of CRS, the patient developed *Candida parapsilosis* sepsis, followed by catastrophic intracranial and pulmonary hemorrhage with refractory shock, leading to death during the same hospitalization. The overall clinical trajectory and escalation of immunomodulatory therapy are summarized in Figure 1.

**Table 1:** Timeline of clinical course.

Date	Event
October 16, 2022	Diagnosis: hypodiploid ALL
November 28, 2022	Achieved remission (AALL1131 protocol)
January 22, 2024	Isolated bone‐marrow relapse
March 17, 2024	Completed FLAG chemotherapy
May 26, 2024	Received CAR T cell infusion
May 27–June 2024	Developed severe CRS → treated with tocilizumab, dexamethasone, anakinra, emapalumab, and supportive care
June 2024	Transient stabilization followed by fatal infectious and hemorrhagic complications

## 3. Discussion

CRS is a common and potentially fatal complication of CAR T cell therapy, driven by elevated levels of cytokines such as IL‐6, IL‐1, and interferon‐gamma. This hyperinflammatory state can lead to vascular leakage, organ dysfunction, and death in severe cases [5, 7]. Effective management often requires a multifaceted approach, including immunomodulators, corticosteroids, and critical care support. Tocilizumab, an IL‐6 receptor antagonist, is typically first‐line for CRS. Anakinra, an IL‐1 receptor antagonist, has shown promise in refractory cases [8]. Although subcutaneous anakinra is more commonly used, IV administration offers enhanced bioavailability and is better suited for critically ill patients. Studies indicate that IV anakinra allows for consistent cytokine suppression, particularly when administered as a continuous infusion [9]. Monteagudo et al. reported high‐dose continuous IV anakinra (up to 2400 mg/day or 2 mg/kg/h) as effective and safe in adults with MAS or secondary HLH. Additionally, pediatric patients (ages 9–17) with severe HLH showed rapid clinical improvement with continuous IV anakinra at doses up to 12 mg/kg/day [10]. Although not widely adopted for CRS in pediatric CAR T cell settings, subcutaneous anakinra is under investigation. In our case, we implemented the continuous IV anakinra infusion protocol used at Regions Hospital. Preparation included mixing 100 mg anakinra in 50 mL normal saline, protected from light, and changed every 8 h to maintain continuous delivery [9]. Emapalumab, a monoclonal antibody against IFN‐*γ*, represents another targeted therapy for refractory CRS. In a case series of 21 pediatric patients undergoing CAR T cell therapy, six received emapalumab after failing conventional interventions. All showed complete leukemia clearance on Day 28, and no additional organ toxicities were reported. Its long half‐life allows for single‐dose use to minimize immune interference [5, 11]. The management of our patient demonstrated the critical role of personalized, dynamic interventions. The coordinated use of tocilizumab, anakinra, dexamethasone, and emapalumab was associated with transient clinical stabilization Despite transient stabilization following aggressive immunomodulatory therapy, the patient subsequently developed fatal infectious and hemorrhagic complications. This case further supports the need for standardized protocols and prospective studies to better define optimal treatment strategies for managing high‐grade CRS in pediatric populations.

## 4. Conclusion

This case illustrates both the potential role and the limitations of intensified immunomodulatory strategies in fulminant pediatric CRS, underscoring the need for prospective studies to define optimal escalation pathways.

## Author Contributions

Abdulrahman Alotaibi contributed to patient care, data collection, interpretation of the clinical findings, and drafting of the initial manuscript. Noura Alajmi contributed to data acquisition and literature review. Mohammed Alnuhait supervised the case report, contributed to the conception and design of the study, critically reviewed and edited the manuscript.

## Funding

No funding was received for this manuscript.

## Disclosure

Noura Alajmi critically revised the manuscript for important intellectual content. Mohammed Alnuhait approved the final version for submission. All authors read and approved the final manuscript and agree to be accountable for all aspects of the work.

## Ethics Statement

This study was approved by the Institutional Review Board of King Abdullah International Medical Research Center (KAIMRC), Riyadh, Saudi Arabia (IRB Number 168225). All data were anonymized in accordance with institutional ethical standards. The IRB waived the requirement for informed consent due to the retrospective nature of the report.

## Consent

No written consent has been obtained from the patient as there is no patient identifiable data included in this case report.

## Conflicts of Interest

The authors declare no conflicts of interest.

## Data Availability

Data supporting the findings of this study are available from the corresponding author upon reasonable request.
